# The STEP-HI Trial: Pragmatic Challenges and Design Issues

**DOI:** 10.1093/geroni/igab046.1143

**Published:** 2021-12-17

**Authors:** Ellen Binder

**Affiliations:** Washington University in St. Louis School of Medicine, Saint Louis, Missouri, United States

## Abstract

Multi-modal interventions present many implementation challenges, especially for studies of frail older adults. The STEP-HI study is an ongoing multi-center, randomized, controlled, double-blinded clinical trial that is evaluating whether six months of topical testosterone therapy combined with a supervised center-based exercise-training program can improve mobility, functional performance, and quality of life after hip fracture, compared to exercise training alone or Enhanced Usual Care. Female hip fracture patients ≥ 65 yrs. old who are living in the community or assisted living are being randomized within 26 weeks of surgical repair for the fracture, and re-evaluated 24 weeks later. This presentation discusses the rationale and study design, and modifications to the protocol in response to challenges, including the COVID-19 pandemic.

